# Tumor of Spinal Cord

**Published:** 1890-07

**Authors:** John M. Parker


### TUMOR OF SPINAL CORD.

By John M. Parker, D.V.S.

On the 5th of February last, a brindled bull terrier, 9 years old, was brought to the dog clinic at the Hospital of the Veterinary Department of McGill University, Montreal.

The subject was well nourished, and in good general health, but for a number of weeks past he had kept up an almost incessant howling, especially during the night, when he was chained up.

He was very lame in the left fore leg; when standing still, or sitting on his haunches, he would lift the leg from the ground, at the same time arching his back and holding his head in a peculiar twisted manner toward the left side. There was a distinct tremor of the muscles of the left shoulder, more marked when sitting on his haunches than at any other time. The twitching was sufficiently well-marked and continuous to be termed choreic. Free passive movements and manipulation of the shoulder caused no pain. There was no atrophy of the muscles of the shoulder. At intervals, especially when any strain came on his collar, he would howl as if suffering the most acute agony.

All the symptoms pointed to a serious derangement of the central nervous system, and as the owner did not wish to have him treated it was decided to destroy him.

*Autopsy*.—Abdominal and thoracic organs normal. On removing the spinal cord, a small tumor was found in the dura mater, situate beneath the cord and toward the left side—between the fourth and fifth cervical vertebræ—firmly attached to the intervertebral substance, and having no connection with the substance of the cord. It was elliptical in form, and measured three-fifths of an inch in length, three-tenths of an inch in breadth, and one-eighth of an inch in thickness. The cord in this region was flattened or hollowed out, the tumor being imbedded in the space thus formed.

Microscopical examination showed the tumor to be a chondrosarcoma with a fibrous capsule, the cartilage cells being scattered irregularly through the substance of the tumor. No alteration in structure of the cord could be made out on section.

The case is interesting from its rarity, and from the difficulty of its diagnosis in the early stages of the disease. Up to 1888 only two cases of excision of tumors in the spinal cord in the human subject had been recorded. Referring to these, *The Medico-Chirurgical Proceedings* for 1888, gives an interesting review of all recorded cases of tumors of the spinal cord. The fifty-eight cases recorded included cases of sarcoma, lipoma, fibroma, tubercle, etc. The causes were various, the most common being exposure to cold, traumatism, mental shock and congenital, more than half being attributed to exposure to cold and traumatism. The total duration of the symptoms averaged two years, the longest time being four and one-half years ; the shortest, six months.

In fifty of the cases the first symptoms noted have been in twenty-eight cases *pain*, in ten cases *motor paralysis*, and in seven cases *sensory paralysis*, while in twenty-three cases only has twitching or tremor been noticed. The usual order of symptoms were *pain*, *motor paralysis* and *sensory paralysis*.

They have invariably been insidious in their attacks, and according to Horsley, "throughout the course of these cases they have almost invariably been diagnosed as rheumatism."

Fortunately, tumors of the spinal cord are easily removed, being confined within a capsule, and rarely implicating the structure of the cord. The greatest antiseptic precautions should be taken.

This is an operation which has not yet been performed upon the lower animals, but with proper precautions I believe it could be performed with perfect safety, and probably with greater prospects of success than in human practice.

